# Supplementary material for: Assessment of nationally representative dietary studies in the Gulf Cooperation Council: a scoping review
Source: PeerJ. 2020 Oct 28;8:e10163. doi: 10.7717/peerj.10163 (PMC7602680; doi:10.7717/peerj.10163)
Supplement: Supplemental Information 1 [file peerj-08-10163-s001.docx]

**Appendix 1** Quality Assessment Scoring Scale for Dietary Studies

|  | Criteria | + / x | Points possible |
| --- | --- | --- | --- |
| **SELECTION** | **Representativeness of the sample** |  | **1 total** |
|  | a) Truly representative of the average in the target population. (all subjects or random sampling) | + | 1 |
|  | b) Somewhat representative of the average in the target population. (non-random sampling) | + | 1 |
|  | c) Selected group of users. | x | 0 |
|  | d) No description of the sampling strategy. | x | 0 |
|  | **Sample size** |  | **1 total** |
|  | a) Justified and satisfactory. | **+** | 1 |
|  | b) Not justified or not satisfactory. | x | 0 |
|  | **Non-respondents** |  | **1 total** |
|  | a) Comparability between respondents and non-respondents characteristics is established, and the response rate is satisfactory (≥60%). | + | 1 |
|  | b) The response rate is unsatisfactory, or the comparability between respondents and non-respondents is unsatisfactory. | x | 0 |
|  | c) No description of the response rate or the characteristics of the responders and the non-responders. | x | 0 |
|  | **Ascertainment of the exposure** |  | **4 total** |
|  | 1. Validated Arabic FFQ plus an additional tool | ++++ | 4 |
|  | 1. Validated Arabic FFQ | +++ | 3 |
|  | 1. Validated FFQ | ++ | 2 |
|  | 1. Non-validated FFQ, but the tool is available or described | + | 1 |
|  | 1. No description of the measurement tool | x | 0 |
| **ADAPTABILITY** | **Adaptability** |  | **1 total** |
|  | a) Incorporation of local foods | + | 1 |
|  | b) No incorporation of local foods | x | 0 |
| **OUTCOME** | **Assessment of the outcome** |  | **3 total** |
|  | a) Use TWO measurements (frequency and quantity) | +++ | 3 |
|  | b) Use times or servings/day or frequency/week | ++ | 2 |
|  | c) Broad frequency scale (always, sometimes, rarely, never) | + | 1 |
|  | d) No description | x | 0 |
|  | **Statistical test** |  | **1 total** |
|  | a) The statistical test used to analyze the data is clearly described and appropriate, and the measurement of the association is presented, including confidence intervals and the probability level (p value). | + | 1 |
|  | b) The statistical test is not appropriate, not described or incomplete | x | 0 |
|  |  |  | **12 TOTAL** |

Cross-sectional study quality:

Excellent studies: 9-12 points

Satisfactory studies: 5-8 points

Unsatisfactory studies: 0-4 points
